# Internet-based exercise interventions for the treatment of depression and anxiety: a systematic review and meta-analysis of randomised controlled trials

**DOI:** 10.1186/s12888-026-08403-4

**Published:** 2026-08-05

**Authors:** Haichang Jia, Zhengyang Mei, Yang Luo, Xintong Mou, Shinan Wei, Jing Huang

**Affiliations:** 1 College of Physical Education, Southwest University, Chongqing, China; 2 College of Pharmacy, China Pharmaceutical University, Nanjing, China

**Keywords:** Internet-based exercise, Depression, Anxiety, Randomised controlled trials, Systematic review, Meta-analysis

## Abstract

**Background:**

Depression and anxiety frequently co-occur and impose substantial public health burdens. Although established treatments are effective, barriers to access and engagement remain. Internet-based exercise may offer a scalable complement to existing treatment options. This review evaluated its effects on depressive and anxiety symptoms.

**Methods:**

This systematic review and meta-analysis was reported in accordance with PRISMA 2020 and registered in PROSPERO (CRD420251131843). We included randomised controlled trials (RCTs) of Internet-delivered structured exercise compared with non-exercise controls and reporting validated measures of depression and/or anxiety. PubMed, Embase, Web of Science, and EBSCOhost were searched, supplemented by manual searches. Risk of bias was assessed using RoB 2. Random-effects meta-analysis was used to calculate standardised mean differences (SMDs) with 95% confidence intervals (CIs).

**Results:**

Thirteen RCTs were included. Eleven trials comprising 1,066 participants contributed to the depressive symptom meta-analysis, and seven trials comprising 607 participants contributed to the anxiety symptom meta-analysis. Internet-based exercise was associated with statistically significant reductions in depressive symptoms (SMD = − 0.53, 95% CI − 0.80 to − 0.26; *I²* = 77.2%) and anxiety symptoms (SMD = − 0.41, 95% CI − 0.65 to − 0.17; *I²* = 48.9%). Most trials were rated as having some concerns regarding risk of bias (11/13), primarily related to outcome measurement. The certainty of evidence was low for depressive symptoms and moderate for anxiety symptoms.

**Conclusion:**

Internet-based exercise may reduce depressive and anxiety symptoms and may serve as a scalable nonpharmacological complement to existing treatments. However, the findings should be interpreted cautiously because of substantial heterogeneity in depressive outcomes and limitations in the certainty of evidence. Further high-quality trials are needed to clarify long-term effects and implementation considerations.

**Registration:**

PROSPERO CRD420251131843.

**Clinical trial number:**

Not applicable.

**Supplementary Information:**

The online version contains supplementary material available at https://doi.org/10.1186/s12888-026-08403-4.

## Background

Depression is a common psychiatric disorder characterised by persistent low mood and loss of interest. It may also involve cognitive symptoms, including difficulties with attention and executive functioning, as well as somatic symptoms [1]. Anxiety disorders, in contrast, are primarily manifested by excessive fear, worry, and autonomic hyperarousal [2, 3]. The two conditions exhibit a high degree of comorbidity and may share overlapping genetic, clinical, and neurobiological features. Anxiety symptoms frequently precede depressive episodes and may influence treatment response [4, 5]. Globally, depression and anxiety are among the leading causes of disability, and their high prevalence and comorbidity often result in more chronic and severe courses [6]. Together, these features underscore the need for accessible and effective interventions for depressive and anxiety symptoms.

Several effective treatment approaches exist for depression and anxiety, including various forms of pharmacotherapy, psychotherapy, and exercise interventions. Antidepressants are established pharmacological treatment options for depression [7, 8]. For the acute treatment of adults with major depressive disorder, Cipriani et al. found that all 21 antidepressants examined were more efficacious than placebo, although efficacy and acceptability varied across drugs [9]. Treatment decisions should therefore consider both expected benefits and individual tolerability. Psychotherapy, represented by cognitive behavioural therapy (CBT), has demonstrated moderate-to-large effect sizes in meta-analyses, yet its relative advantages are inconsistent, and face-to-face delivery remains constrained by limited resources, geographical barriers, and social stigma [10]. Internet-based CBT offers scalability and has shown benefits among both adolescents and adults, although adherence and implementation challenges remain, particularly in unsupported self-guided formats [11–13]. Exercise interventions, including aerobic and resistance training, have been supported by a growing body of meta-analytic evidence demonstrating symptom reduction across diverse populations, with clinical guidelines suggesting that moderate-intensity activity delivered multiple times per week over several weeks may be beneficial [14–16]. Nonetheless, challenges remain regarding methodological inconsistency, uncertainty over optimal parameters for different subgroups, adherence difficulties, insufficient long-term sustainability, and limited accessibility of supervised programmes [17–22]. Collectively, these considerations highlight the value of evaluating Internet-based exercise interventions as accessible and scalable complements to existing treatment options.

The rapid advancement of digital technologies—including smartphones, online platforms, and virtual reality—has facilitated the emergence of Internet-based exercise interventions as practical alternatives to traditional face-to-face programmes, addressing accessibility gaps in healthcare [23, 24]. These interventions are particularly beneficial for individuals facing barriers such as social stigma, transportation difficulties, or geographic isolation, including older adults with mobility limitations [25] and patients living in remote areas [26]. Moreover, the flexibility of online programmes allows individuals with busy schedules to exercise during fragmented periods of free time, catering to the needs of working populations and similar groups [27]. Unlike simple motivational tools, Internet-based interventions provide structured and supervised home exercise prescriptions tailored to individual needs, enhancing adherence through remote monitoring and personalised feedback [28, 29]. The widespread adoption of Internet-enabled devices underscores the scalability of such interventions, with studies reporting high usability and satisfaction across different user groups [30]. Empirical evidence from randomised controlled trials (RCTs) has demonstrated the effectiveness of Internet-based exercise interventions across diverse clinical contexts. For instance, a web-based application with remote technical support improved exercise adherence and functional outcomes in patients with musculoskeletal disorders [31], while online community features reduced attrition rates in Internet-delivered walking programmes, particularly among participants with low social support [24]. Among patients recovering from COVID-19, a home-based telerehabilitation programme led to improvements in anxiety and depression scores [26], and exergaming interventions in older adults showed positive effects on both cognitive and psychological health [32]. Given the accumulating evidence, a systematic review and meta-analysis are warranted to synthesise the effects of Internet-based exercise interventions on depression and anxiety.

Previous systematic reviews examining Internet-based exercise interventions for depression and anxiety have provided valuable insights; however, several methodological limitations warrant further attention. Many reviews adopted broad inclusion criteria and, in some cases, combined exercise with multicomponent interventions such as psychoeducation, physical rehabilitation, or behavioural support, making it difficult to isolate the effect of exercise itself [33, 34]. Across reviews, inconsistent adherence data, lack of blinding, and inclusion of non-randomised studies were also noted, raising the risk of performance and selection bias [34, 35]. To address these methodological challenges, the present review restricted inclusion to RCTs evaluating Internet-delivered structured exercise as an independent intervention component. Studies were excluded when exercise effects could not be isolated from broader multicomponent programmes or when control conditions included structured exercise or additional active psychological components. This review therefore aimed to estimate the effects of Internet-based exercise interventions on depressive and anxiety symptoms while improving the interpretability of the available evidence.

## Methods

This systematic review and meta-analysis was reported in accordance with the Preferred Reporting Items for Systematic Reviews and Meta-Analyses (PRISMA) 2020 [36] and was initially registered in the International Prospective Register of Systematic Reviews (PROSPERO) under the registration number CRD420251131843, with version 1.0 published on 23 August 2025. The initial protocol focused on older adults with mild cognitive impairment (MCI) experiencing symptoms of depression and/or anxiety. Following the initial systematic search conducted on 16 September 2025, the number of eligible randomised controlled trials (RCTs) identified within this narrowly defined population was insufficient to support a quantitative synthesis. The population eligibility criteria were therefore broadened before data extraction and meta-analysis to include participants of any age assessed using validated measures of depression and/or anxiety, irrespective of diagnostic status or cognitive functioning. All retrieved records were subsequently re-screened in full using the amended eligibility criteria. The updated PROSPERO record (version 2.0) was published on 13 October 2025. This post-registration amendment is further acknowledged in the Limitations section.

### Search strategy

Based on medical subject headings and free-text terms, a search was conducted across four databases: PubMed, Embase, EBSCOhost, and Web of Science. Embase was searched via the Embase.com (Elsevier) platform. Searches conducted through EBSCOhost accessed all databases available within our institution’s subscription at the time of the search. Full search strategies for all databases, along with a complete list of the EBSCOhost databases searched, are provided in Supplementary Table S1. To maximise search sensitivity, no controlled-trial filters were applied. Additionally, Google Scholar was manually searched for relevant studies. All searches were run on 16 September 2025, and the search timeframe was from database inception to this date. Following the amendment to the population eligibility criteria, all retrieved records were re-screened using the revised criteria described below.

### Inclusion and exclusion criteria

The inclusion and exclusion criteria were established according to the PICOS framework. Studies were eligible if they met the following criteria: (P) Population: human participants without demographic restrictions; (I) Intervention: We defined Internet-based exercise interventions according to Caspersen et al. (1985), who distinguished between physical activity, defined as any bodily movement produced by skeletal muscles that results in energy expenditure, and exercise, defined as a planned, structured, and repetitive form of physical activity performed to improve or maintain physical fitness [37]. Eligible interventions were required to: (a) deliver structured exercise or prescribed physical activity (e.g., aerobic, resistance, balance, multimodal exercise, yoga, tai chi, dance-based exercise, or exergaming); (b) provide such content via an internet-enabled platform, including mobile applications, websites, videoconferencing, live streaming, pre-recorded videos, or telehealth with remote monitoring or feedback; and (c) require participants to engage in actual exercise/physical activity as part of the programme. Both synchronous (e.g., live online classes) and asynchronous (e.g., app- or web-based guided modules with remote supervision or feedback) formats were eligible, provided that structured exercise was delivered digitally. Interventions consisting solely of physical activity promotion, motivational enhancement, behavioural advice, or educational content without digitally delivered structured exercise sessions were excluded. (C) Comparator: eligible comparators included treatment as usual, routine care, waitlist control, minimal-contact control, or non-exercise digital information/education programmes. Comparators were deemed inappropriate if they contained structured exercise components or additional active psychological therapeutic elements (e.g., online CBT, digital mindfulness, stress-management modules) that could independently affect depression or anxiety, preventing isolation of the exercise effect. This restriction was applied to improve the interpretability of between-group differences as effects associated with Internet-based exercise, while recognising that the intensity and content of eligible non-exercise control conditions could still vary across trials. (O) Outcome: quantitative assessment of depression or anxiety using validated instruments (e.g., PHQ-9, BDI, HAM-D, CES-D, GAD-7, BAI, HAM-A, HADS); (S) Study design: randomised controlled trials.

Studies that did not meet the above PICOS criteria were excluded. This included animal studies, interventions in which the independent effect of Internet-delivered structured exercise could not be isolated, studies without depression- or anxiety-related outcomes, and non-randomised studies or non-primary publications, such as protocols, reviews, conference abstracts, and commentaries.

### Study selection and quality assessment

Dual independent reviewers (HCJ and ZYM) conducted literature screening using EndNote 20.6 reference management software, adhering to predefined eligibility criteria. Following the initial automated duplicate removal by the software, residual redundant entries were eliminated through manual verification. Subsequent title/abstract screening and full-text assessment were independently performed by both researchers. The Revised Cochrane risk-of-bias tool for randomised trials (RoB 2) was used to assess the risk of bias in RCTs, in the following five respects: (1) randomisation process, (2) deviations from intended interventions, (3) missing outcome data, (4) measurement of the outcome, and (5) selection of the reported result. For each eligible study, the overall risk of bias was assessed as either low risk of bias, with some concerns, or high risk of bias [38]. Disagreements during study selection and quality appraisal processes were resolved through consultation with a third investigator (JH).

### Data extraction

Using a data extraction form that included relevant information, two independent researchers collected the following data from each included study: (1) basic information, including the first author, country, and year of publication, (2) participant characteristics, including mean (standard deviation) age, sample size, health status, and baseline depressive symptom scores where reported; (3) intervention characteristics (delivery mode, types, frequency, and duration) and control condition type, and (4) depression- and anxiety-related outcomes.

For all continuous outcomes, post-intervention group-level means, standard deviations, and sample sizes were extracted for both the intervention and control groups. When studies did not report SDs directly, standard conversions recommended by the Cochrane Handbook were applied. In Bossen et al. 2014 [39], SDs were derived from reported 95% confidence intervals. In Chung et al. 2025 [40], mean and SD values were estimated from medians and interquartile ranges using the methods proposed by Wan et al. 2014 [41] and Luo et al. 2018 [42]. All numerical values used in the meta-analysis are provided in Supplementary Table S2 and S3.

### Statistical analysis

Given the expected clinical and methodological heterogeneity across the included trials, including differences in participant characteristics, intervention formats, delivery modes, and outcome measures, pooled effect sizes for both depression and anxiety outcomes were estimated using random-effects meta-analysis with the DerSimonian–Laird method in Stata 18.0 [43]. Standardised mean differences (SMDs; Cohen’s d) with 95% confidence intervals (CIs) were calculated for continuous outcomes and presented in forest plots. Negative SMDs favoured Internet-based exercise. Following Cohen’s conventional benchmarks, SMD magnitudes of approximately 0.20, 0.50, and 0.80 were interpreted as small, moderate, and large effects, respectively; values between these benchmarks were described using intermediate terms, such as small-to-moderate [44]. To account for between-study heterogeneity, 95% prediction intervals (PIs) were also calculated under the random-effects model. Statistical heterogeneity between trials was assessed using Cochran’s *Q* test and the *I²* statistic, with a significance threshold of *p* < 0.10 [45]. A sensitivity analysis was performed for each outcome using a stepwise elimination method to assess whether the pooled results were materially affected by individual studies [43]. Publication bias was assessed by visual inspection of funnel plots and Egger’s test [46, 47]. One of the included trials, Lee et al. 2024 [48], used a multi-arm design with two intervention groups sharing a single control group. Following Cochrane Handbook guidance, only the comparison between the progressive physical activity arm and the control group was included in the meta-analysis to avoid double-counting. The fixed-intensity arm was summarised narratively. Only studies with complete and convertible continuous outcome data contributed to the pooled SMD estimates. All statistical analyses were performed using Stata 18.0.

### Subgroup analyses and meta-regression

In addition to the primary meta-analytic models, subgroup analyses and random-effects meta-regression were conducted to explore potential sources of heterogeneity. As specified in the PROSPERO registration, these analyses were undertaken where sufficient studies were available and focused on broad intervention-related domains. Subgroup and meta-regression analyses were conducted for depression outcomes only. For anxiety, the pooled meta-analysis included only seven trials and demonstrated borderline-moderate, statistically non-significant heterogeneity (*I²* = 48.9%). Given the limited number of studies and the insufficient power to support reliable moderator analyses, no subgroup or meta-regression models were undertaken for the anxiety outcome. For the exploratory analysis of control conditions, comparators were categorised as waitlist or no-contact controls, treatment-as-usual controls, or active non-exercise controls.

The way in which these domains were ultimately operationalised, however—such as the specific duration thresholds or the categorisation of delivery formats—was not predefined in the protocol and was determined after reviewing the available evidence. This revision ensured that all trials could be categorised and is consistent with typical short-term intervention windows (approximately 6–12 weeks) commonly reported in the exercise-for-depression literature (Gordon et al., 2018 [14]). Accordingly, duration-based subgroup analyses were conducted using a ≤ 8-week versus > 8-week categorisation and were treated as exploratory. Subgroup analyses based on participant or study characteristics—such as population type, diagnostic grouping, age group, or risk-of-bias levels—were likewise not included in the PROSPERO protocol. All such exploratory findings should be interpreted with appropriate caution.

## Results

### Literature selection

**Fig. 1:**
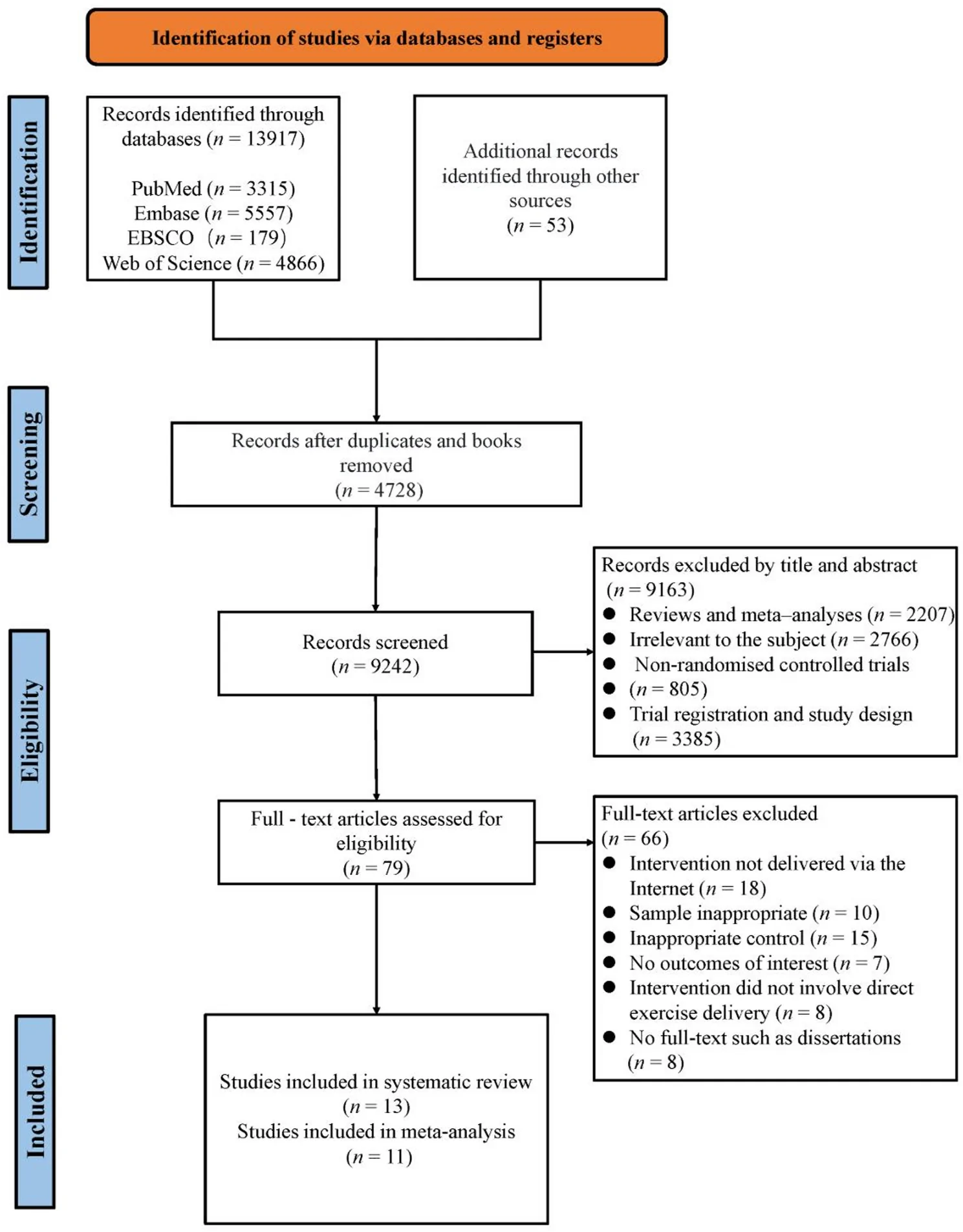
Flowchart of study selection

A total of 13,970 records were identified through database searches and other sources. After duplicate removal, title and abstract screening, and full-text assessment, 13 RCTs were included in the systematic review. Of these, 11 provided sufficient data for quantitative synthesis [16, 39, 40, 48–55], whereas two studies were summarised narratively because convertible post-intervention summary statistics were unavailable (Mascarenhas et al., 2018 [56]; Puterman et al., 2022 [57]). The study selection process and reasons for exclusion are detailed in Fig. 1.

### Characterisation of the study

Thirteen RCTs published between 2014 and 2025 were included following re-screening under the updated eligibility criteria. The studies involved heterogeneous populations, including general adults, older adults, and clinical groups. Intervention durations ranged from 6 to 12 weeks and were delivered through web- or app-based programmes, telehealth or remote-care formats, and live online sessions. Exercise modalities included multimodal programmes, aerobic or high-intensity training, yoga-based interventions, and exergaming. Control conditions were non-exercise comparators, including waitlist, treatment-as-usual, and active non-exercise controls. Detailed study characteristics, outcome measures, and baseline depressive symptom scores are presented in Table 1.

**Table 1:** The main characteristics of the included randomised controlled trials

Included studies	Population		Age [Mean(SD)]	Total/M%	Delivery type	Intervention	Control	Outcome (measure)	Baseline depressive symptom score (as reported)
Badon et al. 2025 (USA)		Postpartum women	T: 32.7 ± 4.8 C: 31.9 ± 4.7	T: 50/NR C: 49/NR	Self-Directed Web / App	Tailored eHealth PA programme Length: 12 weeks Freq: ~3–5 sessions/week (self-directed, monitored) Duration: ~30 min per session	TAU	Depression (PHQ-8) Anxiety (GAD-7)	T: 12.6 ± 2.2 C: 12.6 ± 2.2
Baklouti et al. 2023 (Tunisia)		Older adults	T: 73.07 ± 4.81 C: 72.32 ± 4.38	T: 65/42.86% C: 95/57.14%	Self-Directed Web / App	Web-based Hatha yoga sessions Length: 8 weeks Freq: 3 times/week Duration: 60 min	WL/No-contact	Depression (DASS-21D) Anxiety (DASS-21 A)	T: 7.29 ± 5.28 C: 7.52 ± 5.49
Bossen et al. 2014 (Netherlands)		Osteoarthritis	T: 61.0 ± 5.9 C: 63.0 ± 5.4	T: 100/40% C: 99/30.3%	Self-Directed Web / App	Web-based tailored PA programme Length: 9 weeks Freq: At least 5 days/week (self-monitoring) Duration: ~30 min/day	WL/No-contact	Depression (HADS-D) Anxiety (HADS-A)	T: 4.0 (2.5–5.6) C: 4.2 (2.6–5.9)
Chung et al. 2025 (South Korea)		Chronic respiratory disease (COPD)	T: 65.58 ± 9.22 C: 68.29 ± 7.65	T: 50 /87.8% C: 50 /76.6%	Live / Therapist-Guided	Smartphone app-guided pulmonary rehabilitation (breathing + aerobic + strengthening) Length: 12 weeks Freq: 5 times/week Duration: 40–60 min	Active non-exercise control	Depression (HADS)	T: NR C: NR
Coletta et al. 2024 (USA)		Older adults	T: 70 ± 4 C: 72 ± 5	T: 17/29.4% C: 15/13.3%	Live / Therapist-Guided	Live, therapist-guided online group exercise (strength, balance, mobility) Length: 12 weeks Freq: 3 times/week Duration: 60 min	WL/No-contact	Depression (GDS-SF) Anxiety (GAI-SF)	T: 4.2 ± 2.5 C: 3.5 ± 2.1
Lee et al. 2023 (South Korea)		Community-dwelling older adults	T: 80.39 ± 2.57 C: 79.10 ± 3.90	T: 28/60.7% C: 29/48.3%	Remote Care (Telehealth / Telephone / Gamified–VR / Other)	Home-based exergame using Xbox Kinect (aerobic, balance, muscle-strengthening) Length: 8 weeks Freq: 3 sessions/week Duration: 30–40 min	Active non-exercise control	Depression (GDS-15)	T: 13.21 ± 4.52 C: 13.34 ± 4.75
Lee et al. 2024 (South Korea)		Healthy adults	T: 25.1 ± 4.6 C: 24.7 ± 7.3	T: 19/53% C: 20/50%	Self-Directed Web / App	Mobile-based progressive/fixed PA programme (walking, daily PA goals, app feedback) Length: 12 weeks Freq: 5 times/week Duration: ~30 min/session	WL/No-contact	Depression (PHQ-9) Anxiety (GAD-7)	T: 4.15 ± 4.21 C: 4.85 ± 4.62
Mascarenhas et al. 2018 (USA)		Mothers (general adult women)	T: 30.8 ± 4.6 C: 31.4 ± 4.4	T: 30/NR C: 34/NR	Live / Therapist-Guided	Video exercise groups + PA mobile apps Length: 8 weeks Freq: 5 times/week (weekday mornings) Duration: 30–45 min	WL/No-contact	Depression (PROMIS - D)	T: 48.7 ± 7.6 C: 48.2 ± 6.8
Peng et al. 2018 (China)		Heart failure	T: NR C: NR	T: 49/57.1% C: 49/61.2%	Remote Care (Telehealth / Telephone / Gamified–VR / Other)	Home-based telehealth exercise training (aerobic + resistance, supervised via phone/IM) Length: 8 weeks Freq: 5 times/week Duration: 30–45 min	TAU	Depression (HADS-D) Anxiety (HADS-A)	T: 6.69 ± 0.959 C: 6.65 ± 0.954
Puterman et al. 2022 (Canada)		Healthy adults	T: 41.0 ± 12.1 C: 40.3 ± 12.4	T: 87/13% C: 87/13%	Self-Directed Web / App	HIIT: High-intensity interval training Length: 6 weeks Freq: 4 times/week (for all exercise arms) Duration: 20 min/session	WL/No-contact	Depression (CES-D)	T: 10.4 ± 5.87 C: 10.4 ± 5.54
Seddigh et al. 2023 (Iran)		Type 1 diabetes (adolescents)	T: 14.00 ± 1.03 C: 14.14 ± 1.05	T: 31 / 0% C: 31 / 0%	Remote Care (Telehealth / Telephone / Gamified–VR / Other)	Virtual yoga therapy Length: 8 weeks Freq: 3 times/week Duration: 60 min	TAU	Depression (CDI)	T: 14.64 ± 10.16 C: 13.58 ± 8.10
Tekin et al. 2022 (Turkey)		Chronic neck pain	T: 68.34 ± 4.33 C: 70.34 ± 5.37	T: 132/54.5% C: 123/53.7%	Remote Care (Telehealth / Telephone / Gamified–VR / Other)	Telerehabilitation-based exercise Length: 8 weeks Freq: 3 times/week Duration: 40 min	TAU	Depression (GDS)	T: 16.79 ± 3.42 C: 15.98 ± 3.56
Wadhen et al. 2021 (UK)		Healthy adults	T: 42.7 ± 10.9 C: 42.2 ± 10.2	T: 29 / 0% C: 32 / 0%	Live / Therapist-Guided	Online physiotherapy-based exercise programme Length: 6 weeks Freq: 2 times/week Duration: 45–60 min	TAU	Depression (DASS-21D) Anxiety (DASS-21 A)	T: 6.53 ± 2.98 C: 6.35 ± 3.85

Note: NR = not reported in the original study. Baseline depressive symptom scores are presented in the format reported by the original studies. Values may be reported as mean ± SD, mean (95% CI).

### Quality assessment

**Fig. 2:**
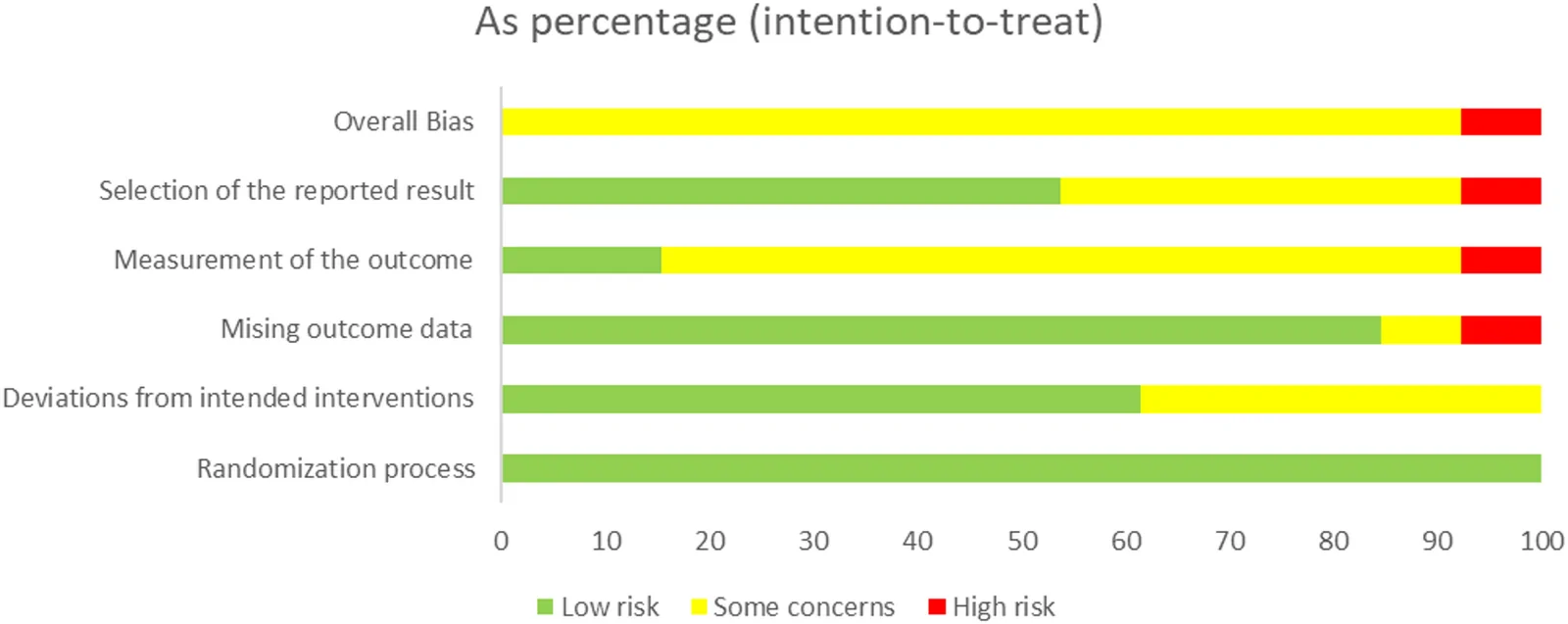
Risk of bias graph

**Fig. 3:**
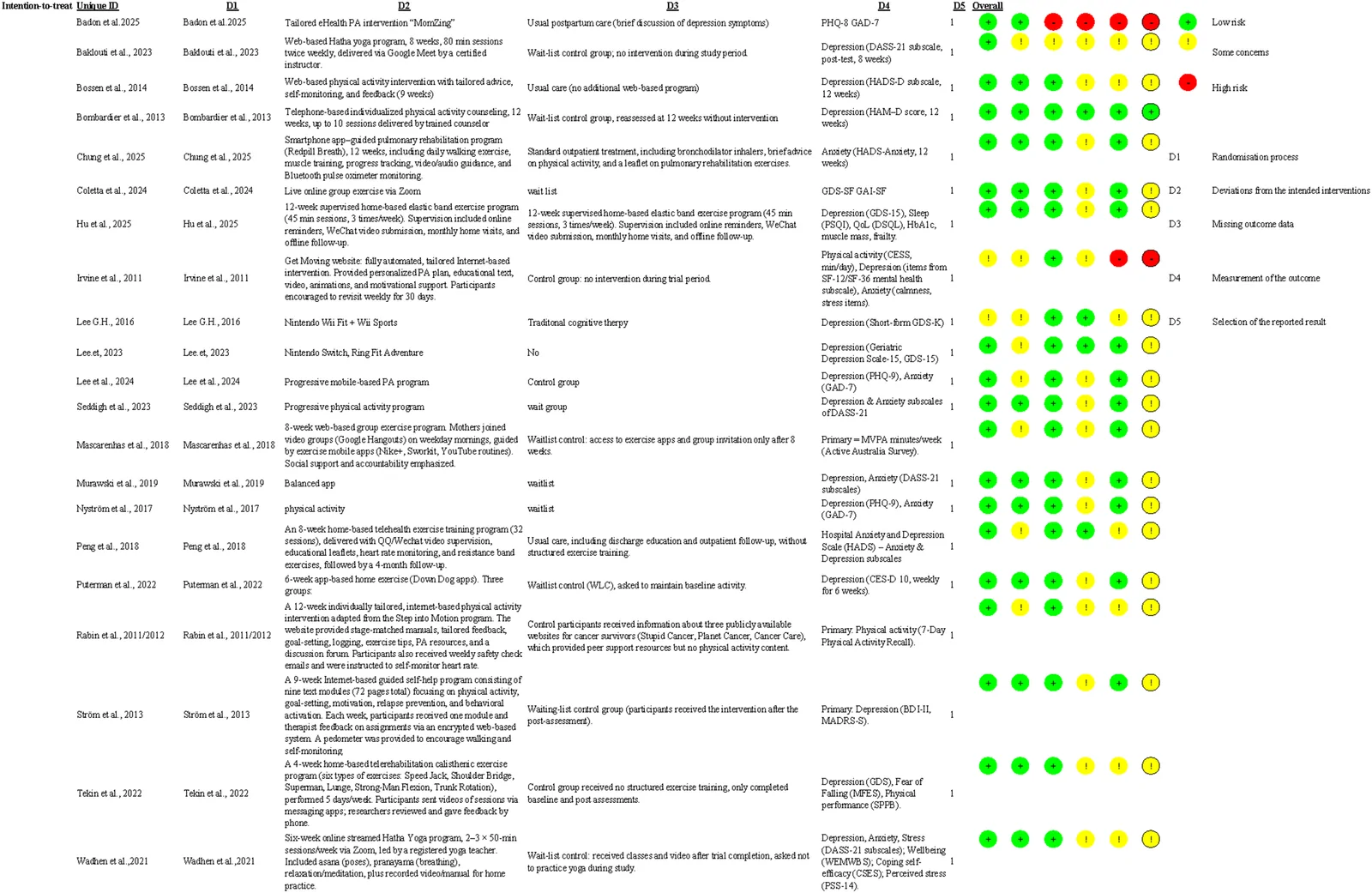
Risk of bias assessment across the five RoB 2.0 domains (D1–D5) for all included trials. Domains: D1 = Randomisation process, D2 = Deviations from intended interventions, D3 = Missing outcome data, D4 = Measurement of outcomes, D5 = Selection of the reported result. Judgement categories: Green = Low risk of bias, Yellow = Some concerns, Red = High risk of bias

Most trials were judged to have some concerns regarding risk of bias (11/13), primarily because blinding was often not feasible and outcomes were assessed using subjective symptom measures. Detailed domain-level assessments are presented in Figs. 2 and 3.

### Meta-analysis

#### Effects of Internet-based exercise on depression

**Fig. 4:**
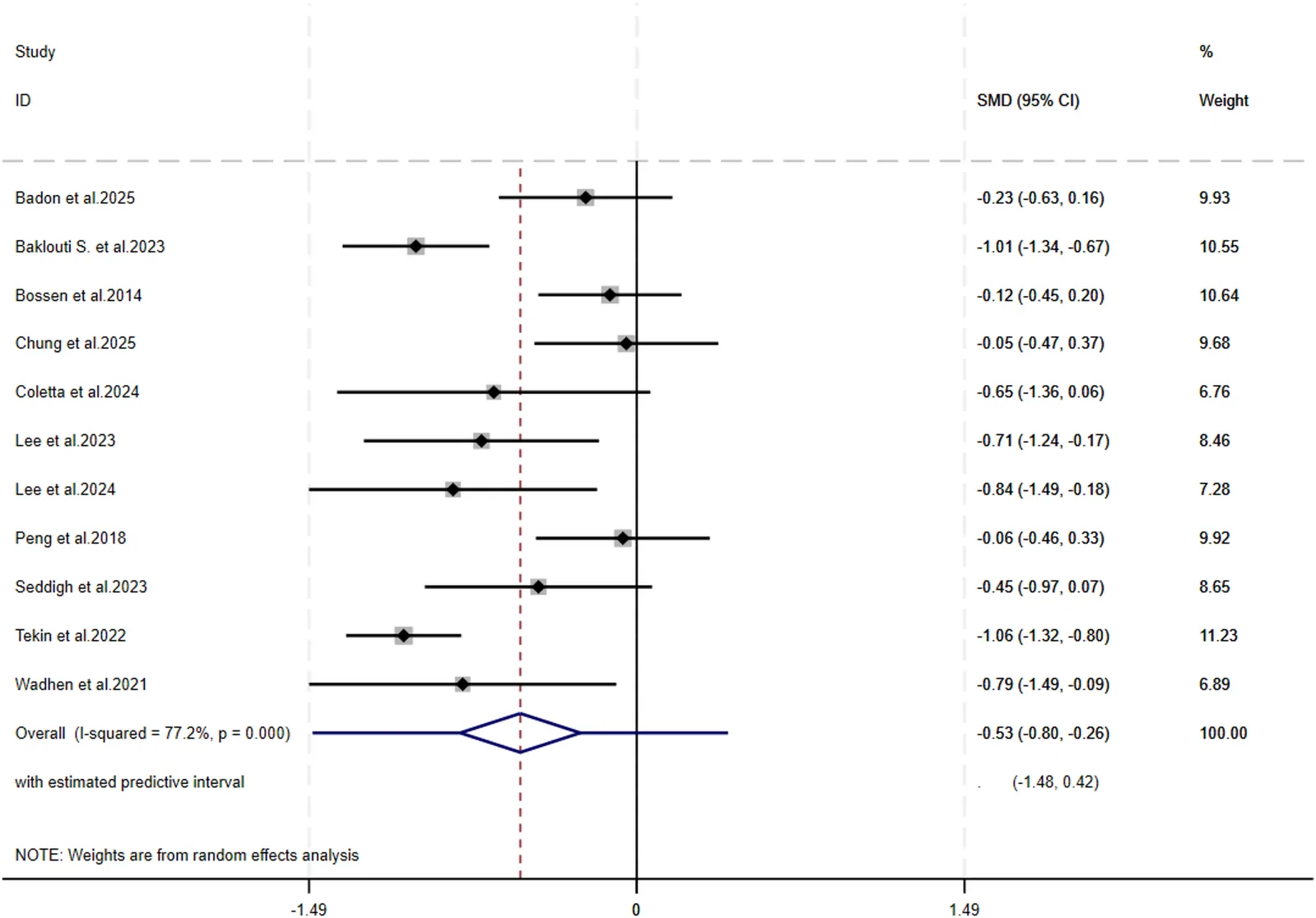
Forest plot of the effects of internet-based exercise on depression

A meta-analysis of 11 RCTs (*n* = 1066 participants) showed a statistically significant reduction in depressive symptoms following Internet-based exercise interventions (SMD = − 0.53; 95% CI − 0.80 to − 0.26; *p* < 0.001), with substantial heterogeneity between RCTs (*I²* = 77.2%; *p* < 0.001). The corresponding 95% PI ranged from − 1.48 to 0.42. A forest plot for depression is presented in Fig. 4.

The group-level means, SDs, and sample sizes used to compute effect sizes are provided in Supplementary Table S2.

#### Effects of internet-based exercise on anxiety

**Fig. 5:**
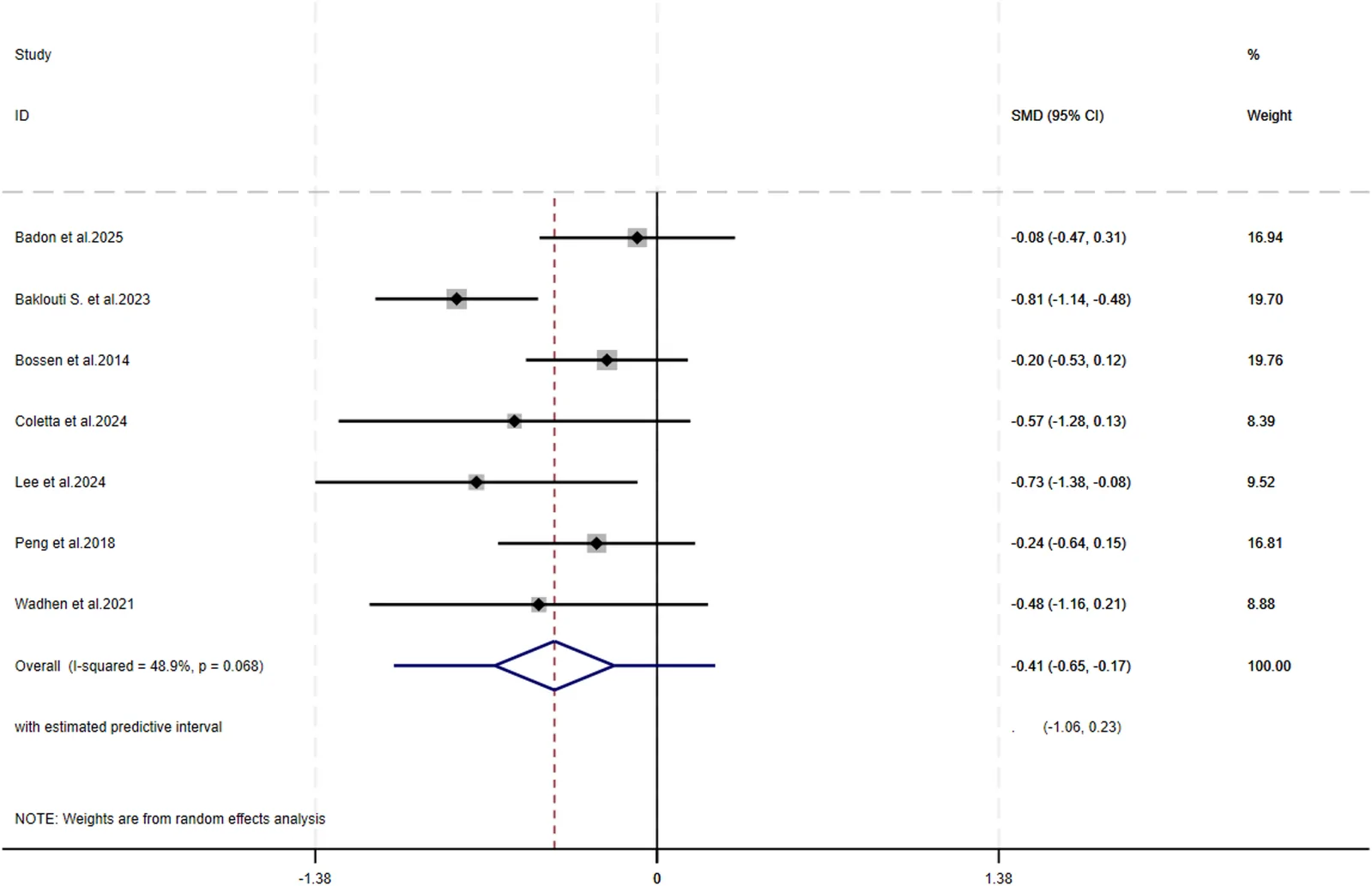
Forest plot of the effects of Internet-based exercise on anxiety

A meta-analysis of 7 RCTs (*n* = 607 participants) showed a statistically significant reduction in anxiety symptoms following Internet-based exercise interventions (SMD = − 0.41; 95% CI − 0.65 to − 0.17; *p* < 0.001), with moderate heterogeneity between RCTs (*I²* = 48.9%; *p* = 0.068). The corresponding 95% PI ranged from − 1.06 to 0.23. A forest plot for anxiety is presented in Fig. 5.

The group-level means, SDs, and sample sizes used to compute effect sizes are provided in Supplementary Table S3. 

#### Narrative synthesis of studies not included in the meta-analysis

Two studies were summarised narratively because their outcome data were not reported in a form that allowed conversion to group-level means and standard deviations. Mascarenhas et al. (2018) [56] provided only adjusted treatment effects without arm-specific post-intervention statistics, while Puterman et al. (2022) [57] reported model-estimated trajectories rather than post-intervention group means. Although neither trial could be pooled quantitatively, both reported improvements in depressive symptoms among participants receiving the exercise interventions.

### Subgroup analyses and meta-regression results

Subgroup analyses and meta-regression were conducted for depressive outcomes only and should be interpreted as exploratory. Control conditions included waitlist or no-contact controls, treatment-as-usual controls, and active non-exercise controls. Neither subgroup analysis (*p* = 0.79) nor meta-regression (β = 0.06, *p* = 0.642) identified a statistically significant association between control condition type and effect size. No statistically significant subgroup differences were observed for delivery/support mode (*p* = 0.91), exercise type (*p* = 0.53), or age group (*p* = 0.85).

Subgroup analyses showed differences according to population type and intervention duration (both *p* < 0.001). However, none of the examined moderators was statistically significant in the meta-regression model. Detailed results are provided in Tables 2 and 3.

**Table 2:** Meta-regression analyses of sources of heterogeneity

Variables	k	β	SE	t	95% CI		*p*-value
					Lower Bound	Upper Bound	
Control condition type	11	0.06	0.12	0.50	-0.27	0.39	0.642
Delivery Mode	11	-0.03	0.16	-0.20	-0.48	0.42	0.851
Exercise type	11	0.04	0.27	0.13	-0.72	0.79	0.899
Population Type	11	0.82	0.34	2.39	-0.13	1.77	0.076
Intervention duration	11	-0.11	0.51	-0.22	-1.51	1.29	0.837
Age Group	11	-0.13	0.20	-0.64	-0.67	0.42	0.555

Note: k = Number of trials; β = Regression coefficient; SE = Standard error; CI = Confidence interval. Moderators were selected based on broad intervention-related domains listed in the PROSPERO protocol. Specific operationalisations and additional study-level factors were determined post hoc; all non-prespecified variables should therefore be interpreted as exploratory

**Table 3:** Subgroup analyses of sources of heterogeneity

Variables	Subgroup	*k*	Trials included	SMD	95% CI		*p*-value (SMD)	Heterogeneity			Group differences (*p*-value)
					Lower Bound	Upper Bound		*I*^*2*^ (%)	*p*-value	Z	
Control condition type	WL/No-contact	4	[39, 48–50]	-0.64	-1.15	-0.13	0.01	79.23	< 0.001	-2.47	0.79
	TAU	5	[16, 52–55]	-0.52	-0.97	-0.07	0.02	82.48	< 0.001	-2.27	
	Active non-exercise control	2	[40, 51]	-0.36	-1.00	0.29	0.28	72.40	0.06	-1.08	
Delivery Mode	Live / Therapist-Guided	3	[40, 50, 55]	-0.43	-0.93	0.08	0.10	52.61	0.12	-1.67	0.91
	Self-Directed Web / App	4	[16, 39, 48, 49]	-0.53	-1.01	-0.06	0.03	82.08	< 0.001	-2.19	
	Remote Care (Telehealth / Telephone / Gamified–VR / Other)	4	[51–54]	-0.58	-1.09	-0.08	0.02	83.46	< 0.001	-2.26	
Exercise type	Single-mode exercise	6	[16, 39, 40, 48, 53, 54]	-0.45	-0.85	-0.05	0.03	83.33	< 0.001	-2.23	0.53
	Multimodal exercise	5	[49–52, 55]	-0.64	-1.04	-0.23	< 0.001	69.22	0.01	-3.08	
Population Type	General Population	7	[48–51, 53–55]	-0.90	-1.07	-0.74	< 0.001	0.00	< 0.001	-10.77	< 0.001
	Patients / Special Populations	4	[16, 39, 40, 52]	-0.12	-0.31	0.07	0.22	0.00	0.92	-1.23	
Intervention duration	≤ 8 weeks	8	[48–55]	-0.71	-0.99	-0.42	< 0.001	65.98	< 0.001	-4.89	< 0.001
	>8 weeks	3	[16, 39, 40]	-0.14	-0.35	0.08	0.22	0.00	0.82	-1.23	
Age Group	< 60 Years	4	[16, 48, 53, 55]	-0.48	-0.76	-0.19	< 0.001	12.24	0.33	-3.30	0.85
	≥ 60 Years	7	[39, 40, 49–52, 54]	-0.52	-0.88	-0.14	0.01	84.90	< 0.001	-2.71	

Note: SMD = Standardised mean difference; CI = Confidence interval; *I*^*2*^ = Heterogeneity index in percentage (range: 0-100%). Only broad intervention-related domains (e.g., intervention type, frequency, duration, delivery format) were outlined in the PROSPERO protocol as potential moderators. All subgroup category definitions were defined post hoc and should be interpreted as exploratory

### Sensitivity analysis

Leave-one-out sensitivity analyses showed that no single study materially changed the pooled estimates for depressive or anxiety symptoms. Detailed results are provided in Table 4.

**Table 4:** Sensitivity analyses for outcomes by omitting individual studies

Outcome	Study omitted	SMD	95% CI	
			Lower bound	Upper bound
Depression	Badon et al.2025	-0.56	-0.86	-0.27
	Baklouti S. et al.2023	-0.47	-0.76	-0.19
	Bossen et al.2014	-0.58	-0.86	-0.30
	Chung et al.2025	-0.58	-0.86	-0.30
	Coletta et al.2024	-0.52	-0.81	-0.23
	Lee et al.2023	-0.51	-0.81	-0.22
	Lee et al.2024	-0.51	-0.80	-0.22
	Peng et al.2018	-0.58	-0.86	-0.30
	Seddigh et al.2023	-0.54	-0.84	-0.24
	Tekin et al.2022	-0.46	-0.71	-0.20
	Wadhen et al.2021	-0.51	-0.80	-0.22
Anxiety	Badon et al.2025	-0.48	-0.73	-0.23
	Baklouti S. et al.2023	-0.27	-0.46	-0.09
	Bossen et al.2014	-0.47	-0.74	-0.19
	Coletta et al.2024	-0.40	-0.67	-0.13
	Lee et al.2024	-0.38	-0.64	-0.12
	Peng et al.2018	-0.45	-0.73	-0.17
	Wadhen et al.2021	-0.41	-0.68	-0.14

SMD, Standardised mean difference; CI, confidence interval

### Publication bias and certainty of evidence

**Fig. 6:**
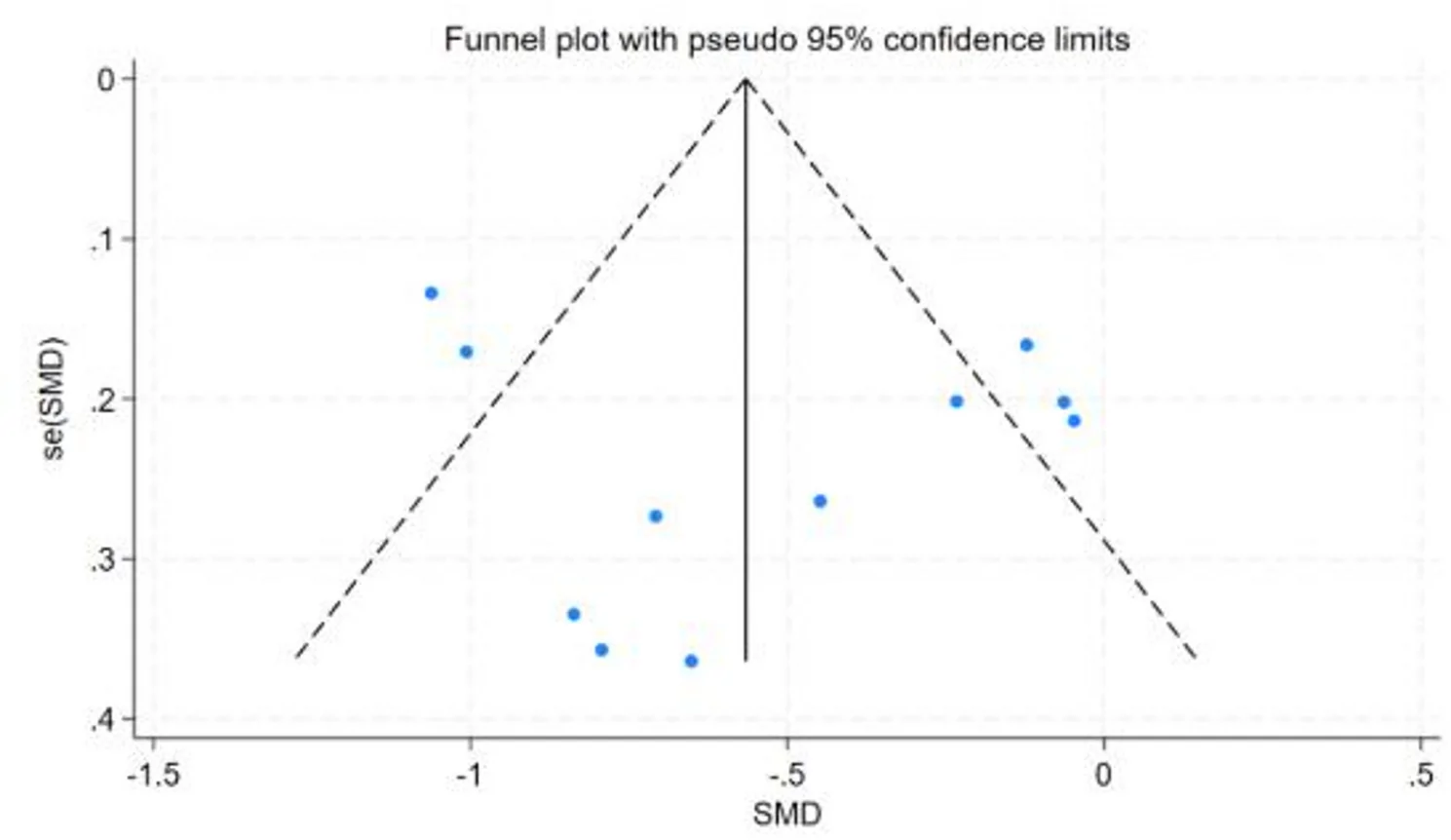
Funnel plot for depression meta-analysis

**Fig. 7:**
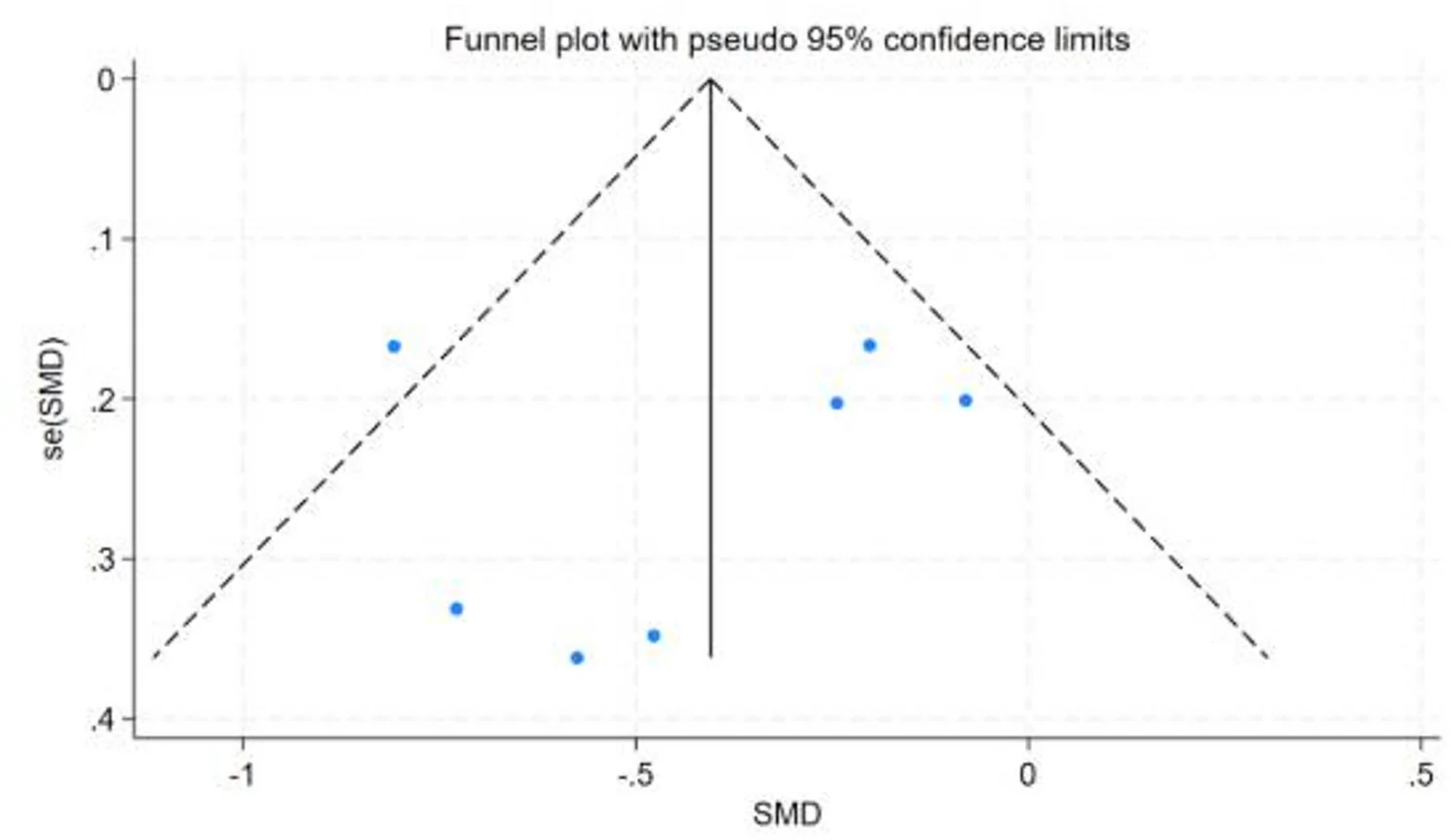
Funnel plot for anxiety meta-analysis

Visual inspection of the funnel plots suggested approximate symmetry for both outcomes (Figs. 6 and 7). Egger’s test did not indicate statistically significant asymmetry for depression (*p* = 0.77) or anxiety (*p* = 0.54).

As shown in the GRADE Summary of Findings (Table 5), the certainty of the evidence was rated as low for depressive symptoms and moderate for anxiety symptoms. For both outcomes, certainty was downgraded by one level for risk of bias because most trials were judged as having some concerns, particularly regarding outcome measurement due to lack of blinding.

**Table 5:** Summary of findings and certainty of evidence (GRADE)

Outcome	№ of studies	Study design	Risk of bias	Inconsistency	Imprecision	Other considerations	Effect (SMD, 95% CI)	Certainty
Depressive symptoms	11 RCTs	Randomised trials	Serious ↓	Serious ↓	Not Serious ↓	None	SMD 0.531 SD lower (0.804 lower to 0.257 lower)	⨁⨁◯◯ Low
Anxiety symptoms	7 RCTs	Randomised trials	Serious ↓	Not Serious ↓	Not Serious ↓	None	SMD 0.414 SD lower (0.654 lower to 0.173 lower)	⨁⨁⨁◯ Moderate

Note: CI: confidence interval; SMD: standardised mean difference

## Discussion

This systematic review and meta-analysis strictly limited inclusion to RCTs and required control groups to consist of non-exercise interventions, thereby improving the interpretability of the observed between-group differences as effects associated with Internet-based exercise. Although the included interventions differed in delivery format (e.g., apps, websites, telehealth, and live online sessions), all were required to deliver structured, exercise-focused content digitally, and interventions in which the independent effect of exercise could not be isolated were excluded. Based on this framework, we synthesised the available evidence to systematically evaluate the effectiveness of Internet-based exercise interventions in alleviating depression and anxiety. On average, Internet-based exercise was associated with reductions in depressive and anxiety symptoms. These pooled estimates should be interpreted cautiously in light of the observed heterogeneity and the methodological limitations identified through the RoB 2 and GRADE assessments. This has substantial public health implications, as the global prevalence and undertreatment of depression and anxiety remain pressing challenges. Although pharmacological and psychotherapeutic treatments are effective, their use is often constrained by side effects, limited accessibility, and social stigma [10, 22]. As a scalable, low-cost, and remotely deliverable intervention, Internet-based exercise represents a promising supplement to existing treatment modalities.

Although the effect sizes were in the small-to-moderate range, their clinical significance should not be overlooked. For depression, the pooled effect size was moderate (SMD = − 0.53; 95% CI − 0.80 to − 0.26), suggesting a statistically significant average reduction in depressive symptoms. For anxiety, the pooled effect size was small to moderate (SMD = − 0.41; 95% CI − 0.65 to − 0.17), and an effect of this magnitude may correspond to perceptible symptom improvement on commonly used anxiety scales such as the GAD-7 or HADS-A, approaching or exceeding minimal clinically important difference (MCID) thresholds in some contexts [12]. Clinically, this implies that some patients may experience perceptible improvements in mood and functioning, particularly among those with mild to moderate depression or anxiety. However, the 95% prediction intervals ranged from − 1.48 to 0.42 for depressive symptoms and from − 1.06 to 0.23 for anxiety symptoms. Because both intervals crossed the null, effects may vary across comparable settings and may not consistently favour the intervention. Therefore, the pooled SMDs should be interpreted as average effects across studies rather than effects expected uniformly across populations and implementation settings. Given the accessibility and scalability of Internet-based interventions, these findings still suggest potential population-level relevance when implemented in appropriate contexts. This pattern aligns with previous findings for non-pharmacological interventions such as traditional exercise and behavioural therapy [20], supporting Internet-based exercise as a statistically effective and potentially clinically meaningful intervention, while underscoring the need for cautious interpretation across heterogeneous settings.

Regarding methodological quality, the 13 RCTs included in this study were generally of acceptable quality. Most trials performed well in terms of randomisation and handling of missing data; however, measurement of outcomes raised some concerns in several studies, which were rated as having “some concerns.” It should be noted that due to the inherent characteristics of exercise interventions, participants are typically aware of whether they are receiving exercise training. As such, strict participant blinding is nearly impossible in this field, representing a limitation of the intervention type rather than a flaw in individual study design [58]. This limitation may introduce potential bias in subjective outcome measures, particularly for depression and anxiety, which rely heavily on self-reported or interviewer-rated scales [38]. Because depression and anxiety were primarily assessed using self-reported symptom scales, lack of blinding may have introduced expectancy effects or differential reporting; therefore, some degree of effect inflation cannot be excluded. This concern is reflected in the GRADE assessment, where certainty was downgraded due to risk of bias related to outcome measurement. Leave-one-out sensitivity analyses indicated that no single study materially influenced the pooled estimates, and publication bias analyses did not reveal clear evidence of asymmetry. However, these findings do not eliminate broader uncertainty arising from risk of bias, heterogeneity, or the limited number of trials.

Exploratory subgroup analyses suggested differences by intervention duration and population type; however, none of the examined moderators was statistically significant in the meta-regression model. These subgroup findings were based on broad post hoc categories and a small number of studies within each subgroup, and should therefore be interpreted cautiously as hypothesis-generating rather than confirmatory. Descriptively, shorter interventions and general population samples were associated with larger pooled effect estimates, but these observations remain tentative, and substantial residual heterogeneity remained unexplained. Control conditions may represent an additional source of clinical heterogeneity. Although exploratory subgroup analysis and meta-regression did not identify a statistically significant association between control condition type and effect size, active non-exercise comparators may yield more conservative estimates by providing additional attention, information, or support. Comparator heterogeneity should therefore be considered when interpreting the pooled effects.

Existing evidence suggests that Internet-based exercise interventions may alleviate depressive and anxiety symptoms through multiple pathways. From a neurobiological perspective, exercise promotes neuroplasticity, with potential mechanisms including increased expression of brain-derived neurotrophic factor (BDNF), which supports structural and functional adaptations in key brain regions such as the hippocampus and prefrontal cortex [59]. Moreover, the anti-inflammatory properties of exercise and its regulatory effects on stress-response systems such as the hypothalamic–pituitary–adrenal (HPA) axis may help lower cortisol levels and further relieve symptoms [60, 61]. At the psychosocial level, exercise interventions may reduce maladaptive cognitive patterns such as rumination by enhancing self-efficacy, providing social interaction opportunities, and fostering mindfulness [62]. Group-based or virtual social engagement may also strengthen perceived social support and mitigate loneliness and helplessness—key psychological dimensions underlying depression and anxiety [63]. The Internet delivery model itself confers unique advantages by improving accessibility and adherence through remote implementation, particularly for individuals facing barriers to in-person interventions [62, 64].

Mechanistic differences across populations also warrant attention. In clinical populations diagnosed with depression or anxiety, exercise may primarily target underlying pathophysiological processes—such as reduced brain volume and systemic inflammation—thereby contributing to symptom alleviation [65, 66]. In contrast, the benefits observed in healthy or subclinical populations may be more preventive in nature, primarily mediated by the maintenance of neuroendocrine homeostasis and enhanced psychological resilience through improved cardiorespiratory fitness [67, 68]. The effects also appear to vary according to exercise parameters; for instance, higher-intensity exercise may be associated with stronger anti-inflammatory and neurotrophic effects, whereas mind–body exercises may primarily engage psychosocial mechanisms [64, 69]. Variation in baseline depressive symptom severity may also have contributed to differences in the observed effects. However, because the included studies used different depression measures and the number of trials was limited, baseline severity was not examined as an additional moderator and its potential influence should be interpreted cautiously. Given current limitations such as reliance on self-reported measures and heterogeneous sample characteristics, future longitudinal studies are needed to clarify causal mechanisms and identify optimal intervention parameters within the digital health framework [70].

From an implementation perspective, Internet-based exercise interventions may improve access to exercise support by reducing geographical and logistical barriers associated with traditional face-to-face programmes [24]. This feature may be particularly relevant for individuals living in remote areas, older adults with limited mobility, and those with restricted access to in-person services. However, this review did not systematically synthesise adverse events or intervention-related withdrawals and therefore cannot draw firm conclusions regarding safety or tolerability. In addition, some studies reported reduced adherence over time, suggesting that maintaining long-term engagement remains an important challenge. Future implementation efforts may benefit from incorporating personalised feedback, social support, and interactive features to support sustained participation.

Although the findings of this study support the potential effectiveness of Internet-based exercise interventions for depression and anxiety, several important research gaps remain. First, the optimal dose, intensity, frequency, and duration of intervention have yet to be established. Although interventions lasting ≤ 8 weeks showed larger pooled effects in the present exploratory analyses, this pattern should not be interpreted as evidence that shorter interventions are inherently more effective. It may also indicate limited sustainability of Internet-based exercise interventions over longer periods, including challenges in maintaining adherence, engagement, or intervention effects over time. Therefore, causal conclusions regarding dose–response relationships cannot be drawn [20], and future multi-arm RCTs with longer follow-up are needed to compare exercise prescriptions and assess maintenance effects. Second, while multimodal interventions showed numerically larger effects than single-mode interventions, no firm conclusions can be drawn because confidence intervals overlapped substantially [12]. Third, current evidence is largely limited to short-term outcomes, and longer follow-up studies are needed to evaluate maintenance effects and relapse prevention. Finally, cultural and socioeconomic factors may contribute to the heterogeneity observed across studies and should be considered when designing and implementing future Internet-based exercise interventions.

## Limitations

Several limitations should be acknowledged. First, the population eligibility criteria were broadened after the initial literature search because the number of eligible RCTs identified in the originally specified MCI population was insufficient to support a quantitative synthesis. Although all retrieved records were re-screened using the amended criteria before data extraction and meta-analysis, this post-registration amendment limits the extent to which the review can be regarded as fully prospective and should be considered when interpreting the findings. Second, moderator analyses were conducted only for depressive outcomes; for anxiety, the limited number of trials and lack of substantial heterogeneity precluded reliable subgroup or meta-regression analyses. Third, subgroup analyses should be interpreted as exploratory because subgroup definitions were set post hoc. Fourth, outcomes were primarily assessed using self-reported symptom scales, and blinding was often not feasible, which may have introduced expectancy effects and contributed to the risk-of-bias and GRADE downgrading. Control conditions also varied in intensity and content, and the limited number of studies within comparator subgroups restricted our ability to determine whether control type modified the observed effects. Fifth, the number of trials eligible for the present meta-analysis was relatively small, and the substantial heterogeneity observed—particularly for depressive outcomes—may further limit the generalisability of the findings. Finally, two eligible trials could not be included in the quantitative synthesis due to non-convertible outcome data, and this review focused primarily on post-intervention outcomes without systematically evaluating longer-term effects or adherence.

## Conclusion

This study, which strictly included RCTs, suggests that Internet-based exercise may independently improve depression and anxiety. However, these findings should be interpreted cautiously given the substantial heterogeneity observed for depressive outcomes and the limited certainty of the current evidence base. Internet-based exercise may still hold potential as a scalable non-pharmacological intervention, but further high-quality, methodologically rigorous trials are needed to confirm its effectiveness, clarify long-term outcomes, and systematically assess safety and feasibility.

## Supplementary Information

Below is the link to the electronic supplementary material.

Supplementary Material 1

## Data Availability

All data generated or analysed in this systematic review and meta-analysis are included in this published article and its supplementary information files. The complete dataset used for meta-analysis (including extracted study characteristics and outcome data) is available in the supplementary tables. Additional information can be obtained from the corresponding author upon reasonable request.

## Abbreviations

BDI-II: Beck Depression Inventory-II
BDNF: Brain-derived neurotrophic factor
CBT: Cognitive behavioural therapy
CI: Confidence interval
GAD-7: Generalised Anxiety Disorder 7-item scale
HADS-A: Hospital Anxiety and Depression Scale–Anxiety subscale
HPA axis: Hypothalamic–pituitary–adrenal axis
I²: I-squared statistic (measure of heterogeneity)
MCID: Minimal clinically important difference
PHQ-9: Patient Health Questionnaire-9
RCT: Randomised controlled trial
RoB 2: Revised Cochrane risk-of-bias tool for randomised trials
SMD: Standardised mean difference
SNRI: Serotonin–norepinephrine reuptake inhibitor
SSRI: Selective serotonin reuptake inhibitor
