# Supplementary material for: Internet-based exercise interventions for the treatment of depression and anxiety: a systematic review and meta-analysis of randomised controlled trials
Source: BMC Psychiatry. 2026 Aug 5;26:864. doi: 10.1186/s12888-026-08403-4 (PMC13732981; doi:10.1186/s12888-026-08403-4)
Supplement: Supplementary file 1 [file 12888_2026_8403_MOESM1_ESM.docx]

**Supplementary Table S1.** **Search strategy**

| PubMed | ((((((((Anxiety disorder[MeSH Terms]) OR (Anxiety[MeSH Terms])) OR (Anxiety disorder[Title/Abstract] OR Anxiety[Title/Abstract] OR Anxi*[Title/Abstract] OR Angst*[Title/Abstract])) OR (Depression disorder[MeSH Terms])) OR (Depression[MeSH Terms])) OR (Depression disorder[Title/Abstract] OR Depression[Title/Abstract] OR Depress*[Title/Abstract] OR Melancho*[Title/Abstract] OR Mood*[Title/Abstract] OR Emotion*[Title/Abstract])) AND (Exercise[Title/Abstract] OR Exerci*[Title/Abstract] OR Physical*[Title/Abstract] OR Training*[Title/Abstract] OR Sport*[Title/Abstract] OR Physical*[Title/Abstract] OR Fitness[Title/Abstract] OR Walking[Title/Abstract] OR Yoga[Title/Abstract] OR Tai Chi[Title/Abstract] OR Dancing[Title/Abstract])) AND (Internet[Title/Abstract] OR Telemedicine[Title/Abstract] OR Mobile health[Title/Abstract] OR Tele-care[Title/Abstract] OR Tele-ICU[Title/Abstract] OR Tele-intensive care[Title/Abstract] OR Telecare[Title/Abstract] OR Telehealth[Title/Abstract] OR Virtual medicine[Title/Abstract] OR eHealth[Title/Abstract] OR mHealth[Title/Abstract] OR Cyber space[Title/Abstract] OR World Wide Web[Title/Abstract] OR Web[Title/Abstract] OR App[Title/Abstract] OR Mobile[Title/Abstract] OR Online[Title/Abstract])) AND ((Randomized controlled trial[Publication Type]) OR (Randomized[Title/Abstract] OR Placebo[Title/Abstract])) |
| --- | --- |
| Embase | ('anxiety disorder':ti,ab,kw OR anxiety:ti,ab,kw OR anxi*:ti,ab,kw OR angst*:ti,ab,kw OR 'depression disorder':ti,ab,kw OR depression:ti,ab,kw OR depress*:ti,ab,kw OR melancho*:ti,ab,kw OR mood*:ti,ab,kw OR emotion*:ti,ab,kw) AND (exercise:ti,ab,kw OR exerci*:ti,ab,kw OR training*:ti,ab,kw OR sport*:ti,ab,kw OR physical*:ti,ab,kw OR fitness:ti,ab,kw OR walking:ti,ab,kw OR yoga:ti,ab,kw OR 'tai chi':ti,ab,kw OR dancing:ti,ab,kw) AND (internet:ti,ab,kw OR telemedicine:ti,ab,kw OR 'mobile health':ti,ab,kw OR 'tele care':ti,ab,kw OR 'tele icu':ti,ab,kw OR 'tele-intensive care':ti,ab,kw OR telecare:ti,ab,kw OR telehealth:ti,ab,kw OR 'virtual medicine':ti,ab,kw OR ehealth:ti,ab,kw OR mhealth:ti,ab,kw OR 'cyber space':ti,ab,kw OR 'world wide web':ti,ab,kw OR web:ti,ab,kw OR app:ti,ab,kw OR mobile:ti,ab,kw OR online:ti,ab,kw) AND ('randomized controlled trial':ti,ab,kw OR randomized:ti,ab,kw OR placebo:ti,ab,kw) |
| EBSCOhost | SU (Anxiety disorder or Anxiety or Anxi* or Angst* or Depression disorder or Depression or Depress* or Melancho* or Mood* or Emotion*) AND SU (Exercise or Exerci* or Physical* or Training* or Sport* or Physical* or Fitness or Walking or Yoga or Tai Chi or Dancing) AND SU (Internet or Telemedicine or Mobile health or Tele-care or Tele-ICU or Tele-intensive care or Telecare or Telehealth or Virtual medicine or eHealth or mHealth or Cyber space or World Wide Web or Web or App or Mobile or Online) AND SU (Randomized controlled trial or Randomized or Placebo) |
| Web of Science | Anxiety disorder or Anxiety or Anxi* or Angst* or Depression disorder or Depression or Depress* or Melancho* or Mood* or Emotion* (Topic) AND Exercise or Exerci* or Physical* or Training* or Sport* or Physical* or Fitness or Walking or Yoga or Tai Chi or Dancing (Topic) AND Internet or Telemedicine or Mobile health or Tele-care or Tele-ICU or Tele-intensive care or Telecare or Telehealth or Virtual medicine or eHealth or mHealth or Cyber space or World Wide Web or Web or App or Mobile or Online (Topic) AND Randomized controlled trial or Randomized or Placebo (Topic) |
| Note: The search timeframe ranged from the inception of each database to September 16, 2025. Searches in all electronic databases (PubMed, Embase, Web of Science, and EBSCOhost) were conducted on September 16, 2025. The search in Google Scholar was also conducted on September 16, 2025. Searches conducted via the EBSCOhost platform included all databases available within the authors’ institutional subscription at the time of the search. Specifically, the following EBSCOhost databases were searched: Academic Search Complete, Applied Science & Technology Source, Arab World Research Source, Art & Architecture Complete, Art & Architecture Source, Business Book Summaries, Business Source Complete, Central & Eastern European Academic Source, CINAHL Complete, Communication Source, eBook Collection (EBSCOhost), EBSCO eClassics Collection, Environment Complete, ERIC, European Views of the Americas: 1493–1750, GreenFILE, Historical Abstracts with Full Text, Humanities Source, Library & Information Science Source, Library, Information Science & Technology Abstracts, Literary Reference Plus, Literary Reference Source eBook Subscription, MEDLINE, Newspaper Source, OpenDissertations, APA PsycArticles, APA PsycINFO, APA PsycTests, Points of View Reference Source, Political Science Complete, Regional Business News, SocINDEX with Full Text, SPORTDiscus with Full Text, and Teacher Reference Center. Teacher Reference Center For Google Scholar, keyword combinations used in the search strategy consisted of (“internet-based exercise” OR “online exercise” OR “web-based physical activity”) AND (“depression” OR “anxiety”) AND (“randomized controlled trial” OR “randomized”). Free-text terms were substituted within the keyword combinations where appropriate. | |

| study | Exp mean | Exp SD | Exp n | Ctr Mean | Ctr SD | Ctr n | Outcome measure used | Timepoint used | Conversions |
| --- | --- | --- | --- | --- | --- | --- | --- | --- | --- |
| Badon et al.2025 | 7.9 | 4.9 | 49 | 9.1 | 5.4 | 50 | PHQ-8 | Post-intervention | No Conversions |
| Baklouti S. et al.2023 | 3.2 | 3.58 | 65 | 10.95 | 9.54 | 95 | DASS-21D | Post-intervention | No Conversions |
| Bossen et al.2014 | 2.4 | 5 | 75 | 3 | 4.82 | 70 | HADS-D | Post-intervention | 95% CI → SD |
| Chung et al.2025 | 9.4 | 6.38 | 41 | 9.75 | 8.18 | 47 | HADS-D | Post-intervention | Median/IQR → mean/SD |
| Coletta et al.2024 | 2.2 | 1.9 | 17 | 3.5 | 2.1 | 15 | GDS-SF | Post-intervention | No Conversions |
| Lee et al.2023 | 10.54 | 3.84 | 28 | 13.55 | 4.62 | 29 | GDS-15 | Post-intervention | No Conversions |
| Lee et al.2024 | 1.52 | 1.61 | 19 | 4.8 | 5.24 | 20 | PHQ-9 | Post-intervention | No Conversions |
| Peng et al.2018 | 6.64 | 0.973 | 49 | 6.7 | 0.924 | 49 | HADS-D | Post-intervention | No Conversions |
| Seddigh et al.2023 | 9.38 | 8.44 | 28 | 12.77 | 6.69 | 31 | CDI | Post-intervention | No Conversions |
| Tekin et al.2022 | 11.49 | 2.66 | 132 | 14.74 | 3.44 | 123 | GDS-30 | Post-intervention | No Conversions |
| Wadhen et al.2021 | 3.06 | 2.81 | 17 | 5.71 | 3.8 | 17 | DASS-21D | Post-intervention | No Conversions |
| Note: Exp = experimental group; Ctr = control group; SD = standard deviation; n = sample size; IQR = interquartile range; CI = confidence interval. | | | | | | | | | |

**Supplementary Table S2. Group-level mean, SD and sample size (n) values used for depression meta-analysis calculations**

**Supplementary Table S3. Group-level mean, SD and sample size (n) values used for anxiety meta-analysis calculations**

| study | Exp mean | Exp SD | Exp n | Ctr Mean | Ctr SD | Ctr n | Outcome measure used | Timepoint used | Conversions |
| --- | --- | --- | --- | --- | --- | --- | --- | --- | --- |
| Badon et al.2025 | 8 | 4.8 | 49 | 8.4 | 5.2 | 50 | GAD-7 | Post-intervention | No Conversions |
| Baklouti S. et al.2023 | 2 | 2.85 | 65 | 6.88 | 7.47 | 95 | DASS-21A | Post-intervention | No Conversions |
| Bossen et al.2014 | 3.1 | 5 | 75 | 4.1 | 4.89 | 70 | HADS-A | Post-intervention | 95% CI → SD |
| Coletta et al.2024 | 1.4 | 2.1 | 17 | 3 | 3.4 | 15 | GAI-SF | Post-intervention | No Conversions |
| Lee et al.2024 | 0.16 | 0.17 | 19 | 0.5 | 0.63 | 20 | GAD-7 | Post-intervention | No Conversions |
| Peng et al.2018 | 6.56 | 0.965 | 49 | 6.77 | 0.743 | 49 | HADS-A | Post-intervention | No Conversions |
| Wadhen et al.2021 | 2.53 | 2.37 | 17 | 3.76 | 2.77 | 17 | DASS-21A | Post-intervention | No Conversions |
| Note: Exp = experimental group; Ctr = control group; SD = standard deviation; n = sample size; CI = confidence interval. | | | | | | | | | |
